# Virological and Epidemiological Features of Norovirus Infections in Brazil, 2017–2018

**DOI:** 10.3390/v13091724

**Published:** 2021-08-30

**Authors:** Sylvia Kahwage Sarmento, Juliana da Silva Ribeiro de Andrade, Marize Pereira Miagostovich, Tulio Machado Fumian

**Affiliations:** Laboratory of Comparative and Environmental Virology, Oswaldo Cruz Institute, Oswaldo Cruz Foundation, Rio de Janeiro 21045-900, Brazil; sylvia.sarmento@ioc.fiocruz.br (S.K.S.); juliana@ioc.fiocruz.br (J.d.S.R.d.A.); marizepm@ioc.fiocruz.br (M.P.M.)

**Keywords:** norovirus, dual genotyping, molecular epidemiology, genetic diversity, viral load, clinical, Brazil

## Abstract

Noroviruses are considered an important cause of acute gastroenteritis (AGE) across all age groups. Here, we investigated the incidence of norovirus, genotypes circulation, and norovirus shedding in AGE stool samples from outpatients in Brazil. During a two-year period, 1546 AGE stool samples from ten Brazilian states were analyzed by RT-qPCR to detect and quantify GI and GII noroviruses. Positive samples were genotyped by dual sequencing using the ORF1/2 junction region. Overall, we detected norovirus in 32.1% of samples, with a massive predominance of GII viruses (89.1%). We also observed a significant difference between the median viral load of norovirus GI (3.4×10^5^ GC/g of stool) and GII (1.9×10^7^ GC/g). The most affected age group was children aged between 6 and 24 m old, and norovirus infection was detected throughout the year without marked seasonality. Phylogenetic analysis of partial RdRp and VP1 regions identified six and 11 genotype combinations of GI and GII, respectively. GII.4 Sydney[P16] was by far the predominant genotype (47.6%), followed by GII.2[P16], GII.4 Sydney[P31], and GII.6[P7]. We detected, for the first time in Brazil, the intergenogroup recombinant genotype GIX.1[GII.P15]. Our study contributes to the knowledge of norovirus genotypes circulation at the national level, reinforcing the importance of molecular surveillance programs for future vaccine designs.

## 1. Introduction

Acute gastroenteritis (AGE) remains one of the major causes of childhood deaths worldwide [1,2]. Among the viruses, norovirus is the leading cause of AGE, affecting people of all age groups. Norovirus is highly transmissible and is estimated to cause one fifth of all AGE cases and over 200,000 deaths globally, with greater impact occurring in children from low- and mid-income countries [3,4]. Especially after the global introduction of the rotavirus vaccine, noroviruses have been considered the most important cause of outbreaks of viral AGE [5,6]. These norovirus-related outbreaks are frequently reported in semi-enclosed and closed settings such as hospitals, cruise ships, long term care facilities, and schools [7].

Noroviruses belong to the family *Caliciviridae* (genus *Norovirus*) and are nonenveloped, positive-sense RNA viruses with a 7.5kb genome that is organized into three open reading frames (ORFs 1–3). ORF1 encodes a polyprotein that is post-translationally cleaved into non-structural proteins. ORF2 encodes the major capsid protein (VP1) with two domains (shell S and protruding P), whilst ORF3 encodes the minor capsid protein VP2 [8]. *Norovirus* genus is currently divided into ten genogroups (GI-GX) and subdivided into over 40 genotypes based on the amino acid sequence of the VP1 protein [9]. While over 30 different genotypes from GI, GII, GIV, GVIII, and GIX can infect humans, a single genotype (GII.4) is responsible for at least 70% of norovirus infections worldwide and has historically been responsible for at least six pandemics reported since the mid-1990s [10,11].

These pandemics are caused by the emergence of GII.4 variants that occurs every 2–5 years replacing the previous dominant strain [11]. The frequent recombination within the ORF1–ORF2 junction region and antigenic variation through amino acid substitutions in the neutralizing epitopes of norovirus capsid protein likely contribute to the emergence of novel strains, thus enabling immune evasion from previous infection [12]. However, the low-level circulation of pre-pandemic strains associated with AGE cases up to 5 years before the onset of the pandemic has been demonstrated [10,13]. Recently, it was shown that changes in host population immunity are also a critical factor for the spread of an antigenically pre-adapted GII.4 variant [14].

While GII.4 noroviruses persist as the dominant strain in circulation worldwide, a number of viruses from other genotypes have emerged in recent years. For instance, a sudden emergence and increase in AGE cases caused by GII.17[P17] norovirus in Asian countries occurred between 2014 and 2015 [15,16], and recently, emergent recombinant genotypes (GII.4 Sydney 2012[P16] and GII.2[P16]) have been detected causing a large number of cases and outbreaks of AGE in different countries [17,18,19]. More recently, a new variant (GII.4 Hong Kong) was reported, although its circulation has been limited to Eurasia since mid-2017 [20].

Brazil has a continental area with a variety of climatic patterns and population conditions that might reflect distinct patterns of viral circulation and genetic diversity. Regional restricted studies have demonstrated the impact of norovirus in Brazil [21,22,23]; however, there is still a gap regarding the burden of AGE caused by norovirus covering different regions and states country-wide. Continuous nationwide surveillance of norovirus-related AGE represents an important tool to understand the epidemiology and disease burden. In this study, we aimed to investigate the incidence of norovirus in Brazil during a two-year period. In addition, we assessed the epidemiological and virological features of norovirus infections, as well as the molecular characterization of genotypes among outpatients with AGE from ten states in three regions. Norovirus was detected and quantified by quantitative RT-PCR (RT-qPCR) from diarrheic stool samples, and dual characterization was performed by sequencing the ORF1-2 junction region. Additionally, we performed the correlation between norovirus shedding among different genogroups and genotypes, age groups and seasonality distribution.

## 2. Materials and Methods

### 2.1. Stool Collection and Ethics Statements

This study included stool samples collected between January 2017 and December 2018 from outpatients (children and adults) with symptoms of AGE, characterized as ≥ three liquid/semi-liquid evacuations in a 24-h period. Diarrheic stool samples were collected from sporadic cases and outbreaks from ten states within three regions of Brazil. Samples were systematically sent together with clinical–epidemiological records to the Regional Rotavirus Reference Laboratory—Laboratory of Comparative and Environmental Virology (RRRL–LVCA). The laboratory is part of the ongoing national network for AGE surveillance and coordinated by the General Coordination of Public Health Laboratories, Ministry of Health (MoH) of Brazil. This study is approved by the Ethics Committee of the Oswaldo Cruz Foundation (FIOCRUZ), number CAAE: 94144918.3.0000.5248 (approval date: 16 August 2018).

### 2.2. Viral RNA Extraction

Viral RNA was purified from 140 μL of clarified stool suspension (10% *w/v)* prepared with Tris-calcium buffer (pH = 7.2). Samples were subjected to an automatic nucleic acid extraction procedure using a QIAamp^®^ Viral RNA Mini kit (Qiagen, CA, USA) and a QIAcube^®^ automated system (Qiagen, Foster City, CA, USA), according to the manufacturer’s instructions. Norovirus RNA was eluted in 60 µL of the elution buffer AVE. The isolated RNA was immediately stored at −80 °C until the molecular analysis. In each extraction procedure, RNAse/DNAse-free water was used as negative control.

### 2.3. Norovirus Detection and Quantification

Norovirus GI and GII were detected and quantified by using a TaqMan-based quantitative one-step RT-PCR (RT-qPCR) with primers and probes targeting the ORF1/2 junction region conserved as previously described [24]. Briefly, RT-qPCR reactions were performed with 5 µL of the extracted RNA in a final volume of 25 µL using the SuperScript™ III Platinum™ One-Step RT-qPCR Kit (ThermoFisher Scientific, Invitrogen Division, Carlsbad, CA, USA) in the Applied Biosystems^®^ 7500 Real-Time PCR System (Applied Biosystems, Foster City, CA, USA). The thermal cycling conditions were carried out as follows: RT step at 50 °C for 60 min, an initial denaturation step at 95 °C for 5 min, and 40 cycles of PCR amplification at 95 °C for 15 s and 60 °C for 1 min. The threshold cycle (Ct) value cut off for a positive result was set at ≤38, and samples that crossed the threshold line showing a characteristic sigmoid curve were regarded as positive. All runs included negative and non-template controls, and a serial diluted (10^1^–10^6^) standard curve (y = −3.43 x + 37.61; efficiency of 95.7%) of a double-stranded DNA fragment containing the target qPCR region (gBlock^®^ Gene Fragment, Integrated DNA Technologies, Coralville, Iowa, USA) to ensure the correct interpretation of the results throughout the study and to estimate viral loads. Norovirus viral loads were expressed as genome copies per gram (GC/g) of stool.

### 2.4. Genotyping and Sequencing

Norovirus-positive samples obtained by RT-qPCR were subjected to conventional RT-PCR for dual genotyping of polymerase and capsid regions. The reactions were performed using the Qiagen One-Step RT-PCR kit (Qiagen, Foster City, CA, USA) with primers Mon 432/G1SKR for GI and Mon 431/G2SKR for GII that target the ORF1/2 junction region [25,26]. The generated amplicons fragments of GI (543 nucleotides (nt)) and GII (557 nt) were purified using the ExoSAP clean-up kit (ThermoFisher Scientific, Waltham, MA, USA) or the QIAquick Gel Extraction Kit (Qiagen, Foster City, CA, USA) and sent to the FIOCRUZ Institutional Platform for Sanger sequencing (PDTIS). Chromatogram analysis and consensus sequences were obtained using Geneious Prime 2020.1.2 (Biomatters Ltd., Auckland, New Zealand). Norovirus genotypes were firstly assigned based on the new nomenclature system using the two norovirus typing tools (https://www.rivm.nl/mpf/typingtool/norovirus and https://norovirus.ng.philab.cdc.gov).

### 2.5. Phylogenetic Analysis

Phylogenetic trees were constructed using the neighbor-joining method for each ORF of GI and GII. The best substitution models were selected based on the corrected Akaike information criterion (AICc) value as implemented in MEGA X v10.1.7 [27]. The model used in this study was Kimura 2-parameter (K2) + G (ORF1 and ORF2) (2000 bootstrap replications for branch support). Norovirus reference sequences were obtained from the National Center for Biotechnology Information (NCBI) database. The sequences obtained in this study were deposited in the GenBank database under the following accession numbers: MZ044561–MZ044563; MZ044943; MZ044968–MZ044975; MZ045410; MZ045426; MZ045595–MZ045608; MZ045718–MZ045736; MZ045797–MZ045832; MZ284846–MZ284863; MZ284950–MZ284951; MZ285603–MZ285731; MZ291635; MZ29163– MZ291642; MZ298338–MZ298359.

### 2.6. Statistical Analysis

Statistical analyses were performed using GraphPad Prism software v8.4.1 (GraphPad Software, San Diego, CA, USA). As appropriate, Mann–Whitney U test, Chi-square, or Fisher test was used to assess the significant difference between norovirus detection rates, years of collecting samples, and age groups, as well as to compare norovirus viral load according to different age groups. A *p*-value <0.05 was considered to be statistically significant.

## 3. Results

### 3.1. Norovirus Epidemiology

**Figure 1:**
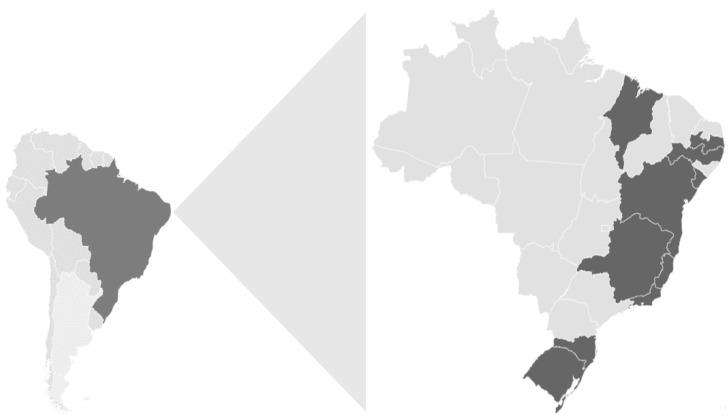
Location of the study areas (states of Espírito Santo, Minas Gerais, Rio de Janeiro, Bahia, Maranhão, Paraíba, Pernambuco, Sergipe, Rio Grande do Sul, and Santa Catarina) within Brazil.

A total of 1546 stool samples were collected from symptomatic outpatients during the two-year period from ten Brazilian states within Southern, Southeastern, and Northeastern regions (Figure 1).

Overall, we detected norovirus in 32.1% of samples (*n* = 497); 25.5% were detected in 2017 and 38.3% in 2018. The norovirus detection rate was significantly higher in 2018 compared to 2017 (*p* < 0.0001). Among these, norovirus GI and GII were detected in 10.9% and 89.1%, respectively, and GI and GII co-infections were observed in 1.4% of samples (*n* = 7) (Table 1).

**Figure 2:**
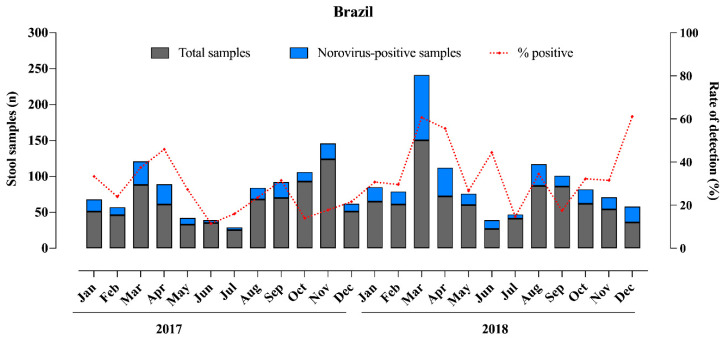
Monthly distribution of tested acute gastroenteritis samples, norovirus-positive samples and norovirus detection rates in Brazil during 2017–2018.

Figure 2 shows the number of stool samples analyzed by month and the number and percentage of norovirus-positive samples. Norovirus was detected year-round and peaked during March and April, although without a marked seasonality. The detection rates varied from 11% to 61% in June 2017 and December 2018, respectively. Concerning seasonal distribution, we observed a higher norovirus circulation during the summer/autumn months compared to winter/spring months; however, no significant difference was observed comparing the detection rates between the two periods (*p* > 0.05).

Norovirus was detected in 52.7% and 47.3% of samples from males and females, respectively, without statistical significance (*p* = 0.5721). In relation to age groups, we detected norovirus across all age groups and detection rates varied from 21.5% in the group of children from 0 to 6 m old to 44.3% in children 6–12 m old, whilst the most affected age group was among children from 6 to 24 m old (Table 2). The detection rate of norovirus among children 6–12 m old was significantly higher compared to other age groups, except for children 12–24 m old (Table 2). Regarding children, adolescents, and adults, included in the >60 m group, we detected norovirus in 16.9% (13/77), 33.3% (12/36), 30.7% (67/218), and 21.6% (8/37) among the age groups of 60 m–13 y, 13 y–20 y, 20 y–60 y, and >60 y old, respectively.

**Figure 3:**
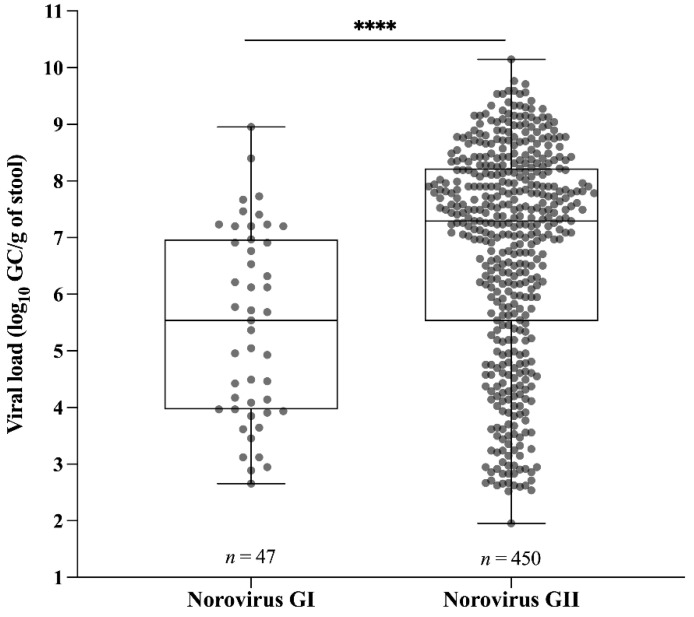
Norovirus viral load expressed as log_10_ genome copies per gram of stool (GC/g) between genogroups I (GI) and GII detected throughout the study. Box-and-whisker plots show the median (the horizontal line) and range of log10 GC/g values. **** *p* ≤ 0.0001.

Concerning norovirus shedding, RT-qPCR results demonstrated that norovirus GI viral load varied from 4.5 × 10^2^ to 8.9 × 10^8^ GC/g of stool, with the median value of 3.4 × 10^5^ GC/g, and GII viral load varied from 9 × 10^1^ to 1.4 × 10^10^ GC/g of stool, with the median value of 1.9 × 10^7^ GC/g. GII-infected patients showed a significantly higher viral load in stool compared to GI-infected patients (*p* < 0.0001) (Figure 3). We also compared norovirus shedding among male (*n* = 262) and female (*n* = 235) patients and no statistical significance was found (median values of 1.4 × 10^7^ GC/g and 1.1 × 10^7^ GC/g of stool for male and female, respectively, *p* > 0.05).

### 3.2. Norovirus Genotyping

**Figure 4:**
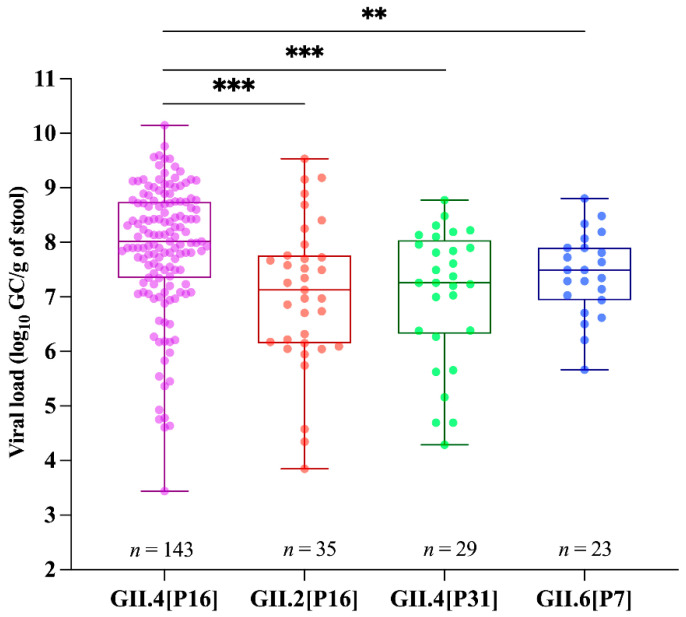
Norovirus viral load expressed as log_10_ genome copies per gram of stool (GC/g) between genogroups I (GI) and GII detected throughout the study. Box-and-whisker plots show the median (the horizontal line) and range of log_10_ GC/g values. *** *p* ≤ 0.001; ** *p* ≤ 0.01.

Dual norovirus genotyping was successfully obtained for 60% (300/497) of positive samples, being 119 sequences from 2017 and 181 from 2018. The most predominant genotype found during the two-year period was GII.4 Sydney[P16] (47.6%; *n* = 143), followed by GII.2[P16] (11.6%; *n* = 35), GII.4 Sydney[P31] (9.6%; *n* = 29), and GII.6[P7] (7.6%; *n* = 23). Samples with low viral concentration (usually above Ct of 32) could not be genotyped. In order to investigate the differences in norovirus shedding among patients infected with different genotypes, we compared viral loads found in stool samples among the four most predominant genotypes. Stool samples from patients infected with GII.4 Sydney[P16] showed significantly higher viral loads (median of 1 × 10^8^ GC/g) compared to GII.6[P7] (median of 3.1 × 10^7^ GC/g), GII.4 Sydney[P31] (median of 1.8 × 10^7^ GC/g), and GII.2[P16] (median of 1.3 × 10^7^ GC/g) (Figure 4).

Among the GI-positive samples (*n* = 47), we sequenced 63.8% (*n* = 30), and six genotypes were characterized. Among those, the most frequent genotype was GI.6[P6], detected in 36.7% (*n* = 11), followed by GI.7[P7] in 33.3% (*n* = 10). The genotypes GI.2[P2], GI.3[P3], GI.3[Pa], and GI.5[P4] were detected in less than 10% of genotyped samples. We also detected one intergenogroup recombinant genotype (GIX.1[GII.P15]) in two samples from Bahia and Santa Catarina states in September and October 2018, respectively.

**Figure 5:**
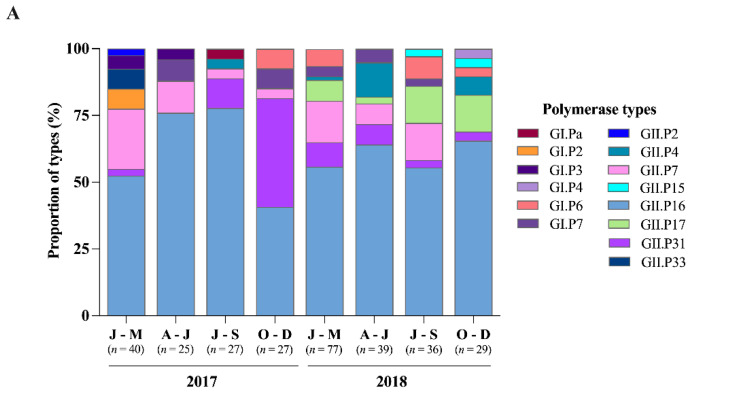

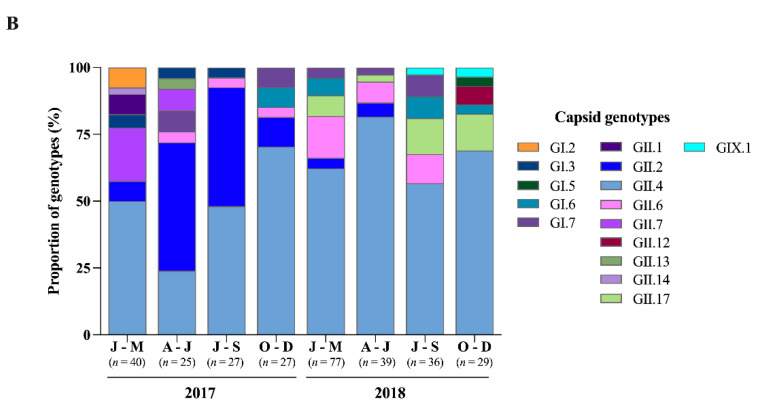
Quarterly distribution of genogroups I, II, and GIX norovirus identified in Brazil between 2017 and 2018 and the proportion of the polymerase (RdRp) types (**A**) and capsid genotypes (**B**) of norovirus strains.

A great diversity of polymerase types and capsid genotypes was found with different proportions over the period (Figure 5). In total, we characterized six GI types (GI.Pa, GI.P2, GI.P3, GI.P4, GI.P6, and GI.P7) and eight GII types (GII.P2, GII.P4, GII.P7, GII.P15, GII.P16, GII.P17, GII.P31, and GII.P33). GII.P16 circulated year-round and was by far the most predominant type detected, followed by GII.P31 and GII.P7 (Figure 5A). Regarding capsid genotypes, five GI (GI.2, GI.3, GI.5, GI.6, and GI.7), nine GII (GII.1, GII.2, GII.4, GII.6, GII.7, GII.12, GII.13, GII.14, and GII.17) and one GIX (GIX.1) were identified. GII.4 was the most predominant genotype, detected in all periods along the two years. GII.2 was the second most common genotype, peaking from April to September 2017, followed by GII.6 and GII.7. It is worth noting that the previously emergent GII.17[P17] genotype was detected only in 2018 (Figure 5B).

**Figure 6:**
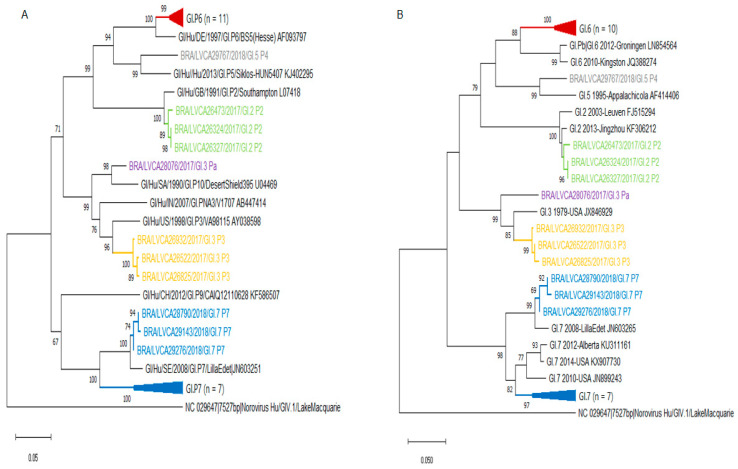
Phylogenetic analyses based on ORF1 (**A**) and ORF2 (**B**) nucleotide (nt) sequences of GI norovirus Brazilian strains. Reference strains were downloaded from GenBank and labeled with their accession number. Sequences obtained are shown as per country followed by the LVCA, internal register number, year, and genotype of collection (i.e., BRA/LVCA26473/2017). Neighbor-joining phylogenetic tree was constructed with MEGA X software and bootstrap tests (2000 replicates), based on the Kimura two-parameter model. Bootstrap values above 60% are given at branch nodes.

We also performed the phylogenetic analyses of polymerase and capsid (VP1) regions of GI and GII norovirus. ORF1 and ORF2 phylogenetic trees confirmed the higher number of recombinant GII genotypes (*n* = 7) compared to GI (*n* = 2). GI.P2, GI.P6, and GI.P7 polymerase types clustered closely with the L07418/GB/1991/Southampton, AF093797/Germany/1997/Berlin, and JN603251/SE/2008/Gothenburg reference strains. The recombinant strain GI.5[P4], detected in 2018, clustered closely with KJ402295/HUN/2013/Baranya strain in the polymerase region (Figure 6).

**Figure 7:**
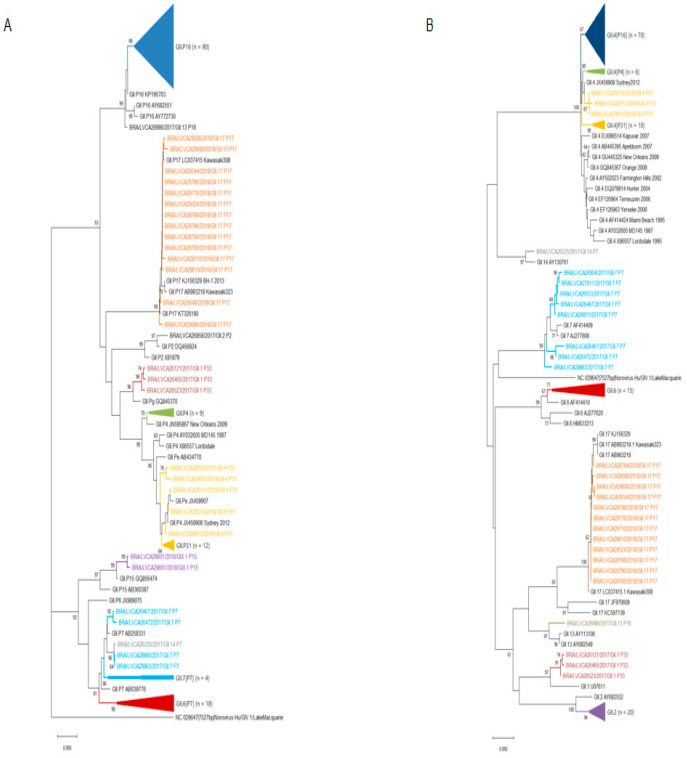
Phylogenetic analyses based on ORF1 (**A**) and ORF2 (**B**) nucleotide (nt) sequences of GII norovirus Brazilian strains. Reference strains were downloaded from GenBank and labeled with their accession number. Sequences obtained are shown as per country followed by the LVCA, internal register number, year, and genotype of collection (i.e., BRA/LVCA26121/2017). Neighbor-joining phylogenetic tree was constructed with MEGA X software and bootstrap tests (2000 replicates), based on the Kimura two-parameter model. Bootstrap values above 60% are given at branch nodes.

Interestingly, the GII.4 capsid strains, detected in association with three different GII types (P31, P16 and P4 New Orleans 2009), were segregated into three different clusters. The type GII.P7 was detected in association with three genotypes (GII.6, GII.7, and GII.14) and segregated into two different clusters. All the GII.17[P17] strains isolated in 2018 in our study clustered within the recently emergent Kawasaki323 reference strain (Figure 7). The intergenogroup recombinant genotype GIX.1[GII.P15] exhibited high similarity (99%) with strains isolated in the US in 2017 (MN227770; MN227776) and Japan in 2020 (LC567100).

## 4. Discussion

Here, we provided results on the incidence of norovirus infections among outpatients with AGE from ten Brazilian states that represent around 100 million inhabitants (almost half of the country’s population), in addition to virological and molecular epidemiological features of these infections. Overall, we detected norovirus in 32.1% of 1546 analyzed samples and GII genotypes, especially GII.4 and GII.2, were by far the most frequently detected throughout the study.

The proportion of norovirus-positive samples found in our study (32.1%) is similar to results found by Safadi et al. [28]. In that study, authors reported an overall detection of norovirus in 23.8% of outpatients’ AGE samples from four countries (Brazil, Chile, Philippines, and Thailand). In Brazil, norovirus predominance was 31% among samples collected between 2015 and 2016 in Belém city, North region [28]. Another study performed in Belém city reported an incidence of norovirus of 24.3% in hospitalized children during a four-year period surveillance study [29]. Similar norovirus detection rates among hospitalized children with AGE were also observed in another two major Brazilian cities: Manaus (35.2%) [30] and São Paulo (28.4%) [31]. In the last decade, norovirus has emerged as the main cause of AGE in countries with the widespread use of rotavirus vaccine, including Brazil [32,33,34]. The worldwide estimated norovirus prevalence is 18% among all AGE cases, 20% among AGE outpatient cases and 17% among hospitalized AGE cases [2]. A large national-based AGE surveillance study conducted in China between 2009 and 2018 revealed norovirus and rotavirus as the leading agents detected in patients aged <5 years, and norovirus was detected in 32.2% of 58,620 analyzed samples [35]. Other studies performed elsewhere have found norovirus prevalence varying from 15% to 21% [33,36,37].

As expected, we found a predominance of norovirus GII (89.1%) compared to GI (10.9%). Several studies performed worldwide have demonstrated the same pattern of GII genotypes predominance among the norovirus infections, with GII detection rates varying between 72.1% and 94.9% [38,39,40]. In Latin American, studies performed in Chile and Bolivia detected GII percentages of 85% and 97.8%, respectively, among patients with AGE [6,41]. In Sergipe state, southeastern Brazil, Santos et al. [42] found similar detection rates of GI (6.4%) and GII (93.6%) among outpatient children <12 years old with AGE, and other studies performed in Brazil also reported the predominance of GII compared to GI among norovirus-positive samples [43,44]. We observed a significantly higher shedding of viral particles per gram of stool in GII-infected patients compared to GI. Even though the pathogenicity and virulence of either GI or GII norovirus genotypes remain unclear [45], a higher concentration in stool samples could indirectly reflect higher contagiousness. Nevertheless, viral load is not the only factor at play in terms of contagiousness since the capacity of immune evade and a better affinity to bind with cellular receptors, for instance, HBGAs, are clearly important co-variables impacting norovirus transmission.

In line with our data, Chan et al. [46] also observed a higher viral load in patients infected with GII compared to GI. In that study, the median viral load values of GI and GII in AGE patients from Hong Kong, China, were 8.4 × 10^5^ and 3 × 10^8^ GC/g of stool, respectively. In another study, viral load data analyzed from outbreak and sporadic cases and asymptomatic controls in the United States and Latin America found that GII-positive specimens had slightly lower Ct values compared with GI-positive samples [47]. It is worth mentioning that interpretation of viral load values in a given population should, however, be very cautious since there is a trend to a gradual decrease of the viral load in stool samples over time during the course of the infection. As the vast majority of the samples included in our study were collected within three days post symptoms onset, we did not evaluate viral load differences considering the course of infection from onset to sample collection, which could be pointed as one limitation of our study. In addition, as we used a DNA standard curve, norovirus viral loads may be overestimated.

Regarding the seasonality, we did not observe a clear seasonal pattern of norovirus circulation. It is well described that in temperate-climate countries, a clearly defined seasonality for norovirus has been described, with the large majority of the clinical cases occurring in winter [18,48,49]. In tropical zones, such as Brazil, norovirus usually circulates throughout the year without a marked seasonal peak [30,42]. In regard to age groups, we observed the highest prevalence of norovirus infections in children aged group from >6 to 24 months old. Several studies have demonstrated the peak of norovirus infection within the same age range [50,51,52,53,54]. Similar to our data, a study performed in Belém, Brazil, from 2012 to 2015 with children hospitalized with AGE, found the majority of norovirus-positive cases (72.7%) among the age group between 6 and 24 months old [29]. The same evidence was reported in a global systematic review showing the higher prevalence of norovirus infections in children less than 2 years old [55]. More recently, the analysis of four cohorts of children <6 years old from four countries (Brazil, Chile, Philippines, and Thailand) demonstrated the highest proportion of norovirus-positive AGE in the 12–23 m age group [28].

Throughout the study, we identified the circulation of several GI and GII genotypes. Among the GI-positive samples, the GI.6 and GI.7 genotypes were the most frequently detected. These genotypes were detected in stool samples of children hospitalized with AGE in several countries worldwide [56,57,58]; however, in our study, they were also detected in adolescents and adults. Alam et al. [59] identified the GI.7 as the most frequent (50%) genotype detected among the GI viruses in hospitalized children in Pakistan. In central-western Brazil, GI.7 was detected in 10% of the genotyped samples from symptomatic and asymptomatic children who attended day care [60].

A larger GII genetic diversity was observed for both polymerase types and capsid genotypes compared to GI. The most predominant genotype detected was GII.4 Sydney[P16] followed by GII.2[P16] and GII.4 Sydney[P31], in agreement with several studies performed world-wide [18,19,54,61,62,63,64,65]. In 2017, we observed a high circulation of the GII.2[P16] at almost similar proportions of GII.4[P16]. From the beginning of 2018 onward, the prevalence of GII.2[P16] diminished and was surpassed by GII.4 Sydney[P16], which was detected in more than 50% of the sequenced samples. It is interesting to note that in 2018, following the spread of GII.4 Sydney[P16], we observed a significantly higher norovirus positivity. Similar evidence of sharp rise and drop in GII.2[P16] infections followed by the GII.4 Sydney[P16] replacement was also observed in Germany [54,63], Australia, and New Zealand [64]. Since 2016, recombinant strains carrying GII.P16, especially GII.4 and GII.2, have caused the majority of norovirus infections world-wide [18,61,66]. By analyzing viral shedding, we found a higher load of GII.4 Sydney[P16] compared to the other three most prevalent genotypes. Previous studies have shown that mutations in the polymerase-encoding region might influence the kinetics and fidelity of the enzyme, suggesting that the mutation rate combined with a higher replication rate might affect the viral fitness of a specific genotype [67,68]. In addition, it was demonstrated that GII.4 viruses have the highest rate of evolution, classified as an “evolving” pattern of diversification, compared to non-GII.4 viruses that were genetically more static [11]. Together, all these features could explain its high transmissibility and widespread circulation.

In addition to GII.4 and GII.2, we also observed a high circulation of the GII.6 genotype combined with the P7 type. This recombinant strain has been circulating at a relatively high frequency in several countries [18,69,70]. Diakoudi et al. [71] reported the majority of GII.6 strains circulating in Italy between 2011 and 2016 was associated with the GII.P7 type (89.2%). Between 2014 and 2016, GII.6 genotype was the second most common genotype detected, after GII.4, accounting for from 2% to 7.1% of the norovirus infections reported by Noronet in Europe and collaborating countries outside Europe [13]. Similar data were also reported in Brazil, where GII.6[P7] was detected in 6.3% of stools samples from AGE children in the Amazonas state [22]. Another study in Brazil detected GII.6[P7] at high frequency in samples of AGE outbreaks that occurred in the Rio Grande do Sul state during eight years (2004–2011) [72].

A great genetic diversity of other genotypes besides GII.4, GII.2, and GII.6 was detected less frequently in our study. Interestingly, the recently emergent genotype GII.17[P17] was detected only in 2018. Sequences isolated clustered within the reference Kawasaki_323 from Japan, represented by viruses that later emerged in the winter season of 2014–2015 in Asian countries [15,73,74] and subsequently spread to countries outside Asia, indicating that GII.17 could possibly replace GII.4 on a global scale [75,76]. In Brazil, during a four-year surveillance study from 2015 to 2016, GII.17[P17] was detected in 12.6% of AGE stool samples in the Northern region [77], and a previous study from our group detected this genotype in three distinct Brazilian states [78]. We detected the uncommon intergenogroup recombinant GIX.1[GII.P15] in two AGE sporadic cases from two states in 2018. This genotype was reported during an outbreak investigation that occurred in a cross-border travel group with symptoms of gastroenteritis travelling from Thailand to China, where GIX.1[GII.P15] was detected in 8.6% of cases and also co-detected with three additional genotypes in one patient [79].

Our study included data from ten Brazilian states, representing around 100 million inhabitants (almost half of the Brazilian population). However, it is worth mentioning that the variability in reporting and collecting AGE cases by states generates surveillance biases. Another limitation is that we did not search for nt mutations in the antigenic epitopes within the P2 subdomain in order to monitor GII.4 evolution over time. Nevertheless, future studies are planned to investigate the genetic characterization and evolution of GII.4 viruses based on the P2 nt sequence or the entire VP1capsid protein.

In conclusion, during the period of study, we detected 32% of norovirus positivity and besides a great diversity of genotypes circulating in Brazil, GII viruses, specifically GII.4 and GII.2, were dominant over the period. Currently, norovirus vaccines are in clinical trials (phase I and II); however, these vaccine candidates face the challenge of an unclear definition of cross-protection immunity among the different genotypes. Country-based epidemiological and molecular surveillance programs can assist in global decision-making regarding vaccines design and implementation policies. Moreover, as new genotypes or variants may emerge in any part of the world and spread quickly, continuous surveillance programs are necessary to follow epidemiological and evolutionary trends of norovirus infections.

## Figures and Tables

**Table 1:** Number of norovirus-positive fecal samples and percentage of genogroup I (GI) and GII through laboratory-based surveillance by region and state in Brazil during 2017 and 2018.

Region/ State	N° of Fecal Samples–Positive/Tested (%)			*p*-Value ^1^ (Chi-Square Test)
	Total % of GI–GII	2017	2018	
**Southeastern**	94/274 (34.3) 12.8–87.2	27/106 (25.5)	67/168 (39.8)	0.0144
Espírito Santo		6/24	22/56	
Minas Gerais		11/52	38/75	
Rio de Janeiro		10/30	7/37	
**Northeastern**	149/613(24.3) 7.4–92.6	64/361 (17.7)	85/252 (33.7)	<0.0001
Bahia		22/74	26/98	
Maranhão		17/55	3/8	
Paraíba		3/25	0/37	
Pernambuco		8/150	31/68	
Sergipe		14/57	25/41	
**Southern**	254/659 (38.5) 12.2–87.8	99/278 (35.6)	155/381 (40.6)	0.1865
Rio Grande do Sul		33/131	50/168	
Santa Catarina		66/147	105/213	
**Total**	497/1,546 (32.1) 10.9–89.1	190/745 (25.5)	307/801(38.3)	<0.0001

^1^*p*-values were calculated comparing norovirus frequency of detection between the years of 2017 and 2018.

**Table 2:** Number of tested and norovirus-positive fecal samples through laboratory-based surveillance by age group in Brazil during 2017–2018.

Age Group (Months)	N° of Fecal Samples–Positive/Tested (%)			*p*-Value ^1^ (Chi-Square Test)
	2017	2018	Total	
0–6	28/118 (23.7)	24/123 (19.5)	52/241 (21.5)	<0.0001
>6–12	47/122 (38.5)	66/133 (49.6)	113/255 (44.3)	-
>12–24	54/174 (31.0)	104/203 (51.2)	158/377 (41.9)	0.9742
>24–60	30/164 (18.3)	44/141 (31.2)	74/305 (24.2)	<0.0001
>60	31/167 (18.5)	69/201 (34.3)	100/368 (27.1)	<0.0001

^1^*p*-values were calculated between the age group of >6–12 and each other.

## Data Availability

The data presented in this study are available on request from the corresponding author. The data are not publicly available due to privacy and ethical restrictions. Nucleotide sequences are available in GenBank database under the deposited numbers cited within the manuscript.
